# Acoustic spin Hall-like effect in hyperbolic metamaterials controlled by the helical wave

**DOI:** 10.1038/s41598-018-29359-w

**Published:** 2018-07-24

**Authors:** Fangfang Ju, Ying Cheng, Xiaojun Liu

**Affiliations:** 1 Key Laboratory of Modern Acoustics, Department of Physics and Collaborative Innovation Center of Advanced Microstructures, Nanjing University, Nanjing, 210093 China; 2 State Key Laboratory of Acoustics, Institute of Acoustics, Chinese Academy of Sciences, Beijing, 100190 China

## Abstract

Because of the spin-less nature of sound, acoustic helical wave with different helical directions can be taken as a “spin-like” degree of freedom. In this Letter, we examine the pseudospin-orbit coupling effect in acoustics when an acoustic helical wave emitter interacts with the acoustic hyperbolic metamaterial (AHMM). The acoustic helical wave emitter is situated at the boundary of the AHMM, which gives rise to the unidirectional excitation with the trajectory controlled by the helical directions, and hence the acoustic spin Hall-like effect (ASHE) is observed. The ASHE is further demonstrated for the string-type and the membrane-type AHMM based on the hyperbolic dispersion. The reported ASHE paves a new way to exploiting signal routing and unidirectional excitation controlled by the helical directions of the acoustic helical wave.

## Introduction

Metamaterials are artificially engineered media made from a periodic spatial modulation of acoustic subwavelength building blocks. Through careful design of the material’s composition, size, structure and mutual orientations, many intriguing properties have been realized which cannot be found in nature materials. Over the past decades, there are various structures proposed to realize versatile properties, such as a high-density solid core with soft coating which is the first locally resonant sonic unit to realize the negative dynamic mass density^1^, Helmholtz resonators which consist of a short neck and cavity^2^, ultraslow-fluid-like particle which exhibits intense Mie resonances^3^, coiling up space using curled channels of subwavelength cross section^4^, etc. Such remarkable structures have enabled sound propagation in unprecedented manners, including negative refraction^5^, super-resolution imaging^6^, invisibility cloaking^7,8^, and zero-index waveguide^9,10^. However, most applications of metamaterial concentrate on the classical physics in the low frequency range and the wavelength is too large to emerge quantum-like behaviors.

In addition to the quantum-like effects in acoustics studied in the very high frequency range, the close analogies between the classic physical phenomena in acoustic metamaterials and the quantum effects in electronics provide us an ideal platform to study the quantum-like effects in acoustics. Many intriguing properties have been demonstrated which are in close analogy with quantum effects in the electronics, such as Parity-time symmetric acoustics^11,12^, robust pseudospin-dependent one-way edge sound transport in acoustic topological insulator^13–18^, Dirac-like cone to realize acoustic double zero refractive index metamaterials^19^, topological valley transport of sound^20^, an analogue of Zitterbewegung effects in surface phononic graphene^21^, etc. It is noted that there are also many studies on the optical quantum-like effects as analogies to the quantum effects in the electronics, which give us a more direct route to the realization of the acoustic quantum-like effects since there are many similarities in optical waves and acoustic waves. For example, photonic spin Hall effect in hyperbolic metamaterials is in close analogy to the spin Hall effect for electrons where the different polarizations of photons play the role of “spin”^22^. With the inspiration of that work, in this letter, we introduce the acoustic spin Hall-like effect (ASHE) in the acoustic hyperbolic metamaterial (AHMM). AHMM is a special class of acoustic metamaterial deriving their name from the unique form of the equifrequency contour (EFC) which is hyperbolic instead of circular as in conventional materials. Thus, AHMMs can transmit sound with very high wavevector components which come to be evanescent in conventional materials. We exemplify the AHMM by multiple arrays of thin string/membrane. The acoustic helical wave with different helical directions is taken as an acoustic “spin-like” degree of freedom, which can be realized by the array of the acoustic point source or the recently proposed acoustic metamaterials giving rise to the orbital angular momentum^23–25^.

## Results

**Figure 1:**
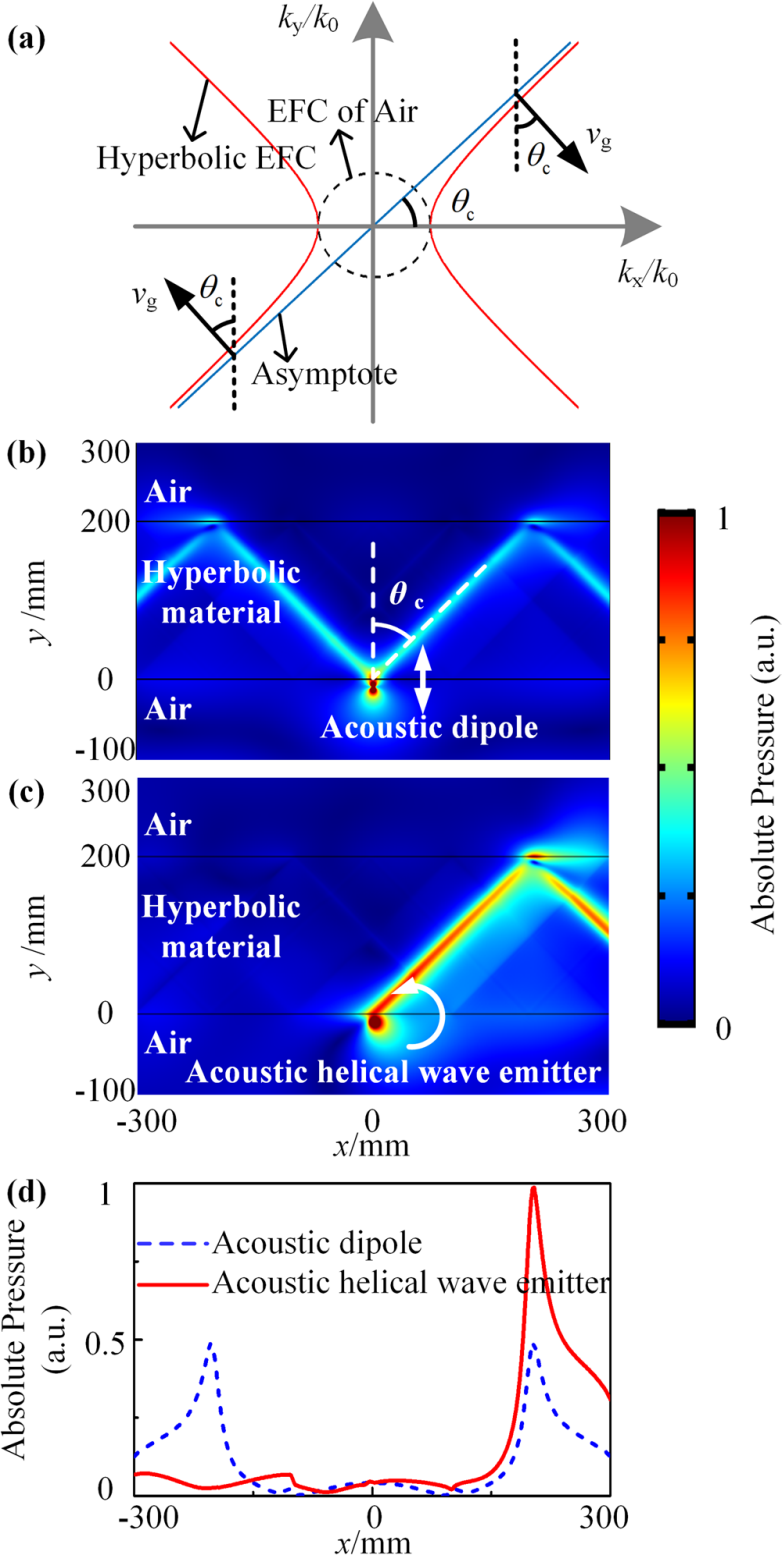
**(a)** Equifrequency contour of *k*_x_ − *k*_y_ plot in the AHMM with hyperbolic dispersion (*ρ*_x_ = 1.39 kg/m^3^, *ρ*_y_ = −1.39 kg/m^3^, *K* = 152 KPa). Absolute pressure distribution of **(b)** acoustic dipole and **(c)** acoustic helical wave emitter radiating in the vicinity (at 10 mm distance from the interface) of an AHMM slab sandwiched by air (See Supplementary Movie 1 for dynamic view). **(d)** Absolute pressure along the cutline *y* = 200 mm of **(b)** and **(c)**.

First, we demonstrate the unidirectional excitation in the AHMM. Figure 1(a) illustrates the EFC of *k*_x_ − *k*_y_ plot in the AHMM. In two-dimensional (2D) scenarios, the acoustic waves in the anisotropic medium are governed by the density ${\rho }_{\theta }$, which depends on the propagation direction^22^:

1 $$\frac{{k}_{{\rm{\theta }}}^{2}}{{\rho }_{{\rm{\theta }}}}=\frac{{k}_{{\rm{x}}}^{2}}{{\rho }_{{\rm{x}}}}+\frac{{k}_{{\rm{y}}}^{2}}{{\rho }_{{\rm{y}}}}$$

where $\theta$ is the angle between the wave vector and the *x* axis. ${\rho }_{{\rm{x}}}$ (${k}_{{\rm{x}}}$), ${\rho }_{{\rm{y}}}$ (${k}_{{\rm{y}}}$) and ${\rho }_{{\rm{\theta }}}$ (${k}_{{\rm{\theta }}}$) are the effective mass density (wave vector) in the *x* direction, *y* direction, and $\theta$ direction, respectively. For the AHMM $({\rho }_{{\rm{x}}} > {\rm{0}},{\rho }_{{\rm{y}}} < 0)$, ${\rho }_{{\rm{\theta }}}$ diverges at the angle ${\tan }^{2}{\theta }_{{\rm{c}}}=|\frac{{\rho }_{{\rm{y}}}}{{\rho }_{{\rm{x}}}}|$ which is the direction of the asymptote of the hyperbolic EFC. Thus, the density of acoustic states (DOS) at this angle is infinite. As the group velocity must lie normal to the EFC, all acoustic waves will propagate forming a cone with an opening angle of ${\theta }_{{\rm{c}}}$ [see Fig. 1(a)]. i.e., the propagation channel in the AHMM is automatically selected.

As the helical wave can be expressed in the form of a dipolar moment $${\bf{p}}=[1,\pm i]$$, we consider a 2D acoustic emitter with a dipolar moment $${\bf{p}}=[{p}_{{\rm{x}}},{p}_{{\rm{y}}}]$$, and then the pressure field can be written as $$P(x,y)=i{k}_{0}{\rho }_{0}{c}_{0}{\bf{p}}\cdot $$$$\nabla g(|\overrightarrow{r}-{\overrightarrow{r}}_{0}|)$$, where $${k}_{0}$$ is the wave vector; $${\rho }_{0}$$ is the density; $${c}_{0}$$ is the sound speed of the medium; $${r}_{0}$$ is the position of the acoustic emitter^26^.$$g(|\overrightarrow{r}-{\overrightarrow{r}}_{0}|)=\frac{{{\rm{e}}}^{{ik}|\overrightarrow{r}-{\overrightarrow{r}}_{0}|}}{4{\rm{\pi }}|\overrightarrow{r}-{\overrightarrow{r}}_{0}|}$$, and $$r=\sqrt{{(x-{x}_{0})}^{2}+{(y-{y}_{0})}^{2}}$$. Since the angular spectrum representation of the function $$\,g(r)$$ is derived that $$\frac{{{\rm{e}}}^{{ikr}}}{4{\rm{\pi }}r}=\frac{i}{2{\rm{\pi }}}{\int }_{-\infty }^{\infty }\,\frac{{{\rm{e}}}^{i{k}_{{\rm{x}}}x+i{k}_{{\rm{y}}}|y|}}{{k}_{{\rm{y}}}}{\rm{d}}{{k}}_{{\rm{x}}}$$ and the angular spectrum representation of the pressure field $$\,P(x,y)$$ can be written as $$P(x,y)={\int }_{-\infty }^{\infty }P({k}_{{\rm{x}}},y){{\rm{e}}}^{i{k}_{{\rm{x}}}{x}}{\rm{d}}{k}_{{\rm{x}}}$$, it can be derived as

2 $$P({k}_{{\rm{x}}},{y})=-\,\frac{i{k}_{0}{\rho }_{0}{c}_{0}}{2{\rm{\pi }}}(\frac{{k}_{{\rm{x}}}}{{k}_{{\rm{y}}}}{p}_{{\rm{x}}}\pm {p}_{{\rm{y}}}){{\rm{e}}}^{i{k}_{{\rm{y}}}|y-{y}_{0}|}$$

where ${k}_{{\rm{y}}}\,=\,\sqrt{{k}_{0}^{2}-{k}_{{\rm{x}}}^{2}}$, the angular frequency $\omega ={k}_{0}{c}_{0}$, and the phasor notation $P(\overrightarrow{r},t)=\mathrm{Re}[P(\overrightarrow{r}){{\rm{e}}}^{-i{\rm{\omega }}t}]$^27,28^. The upper (lower) sign in Eq. (2) applies to $y > {y}_{0}(y < {y}_{0})$. Here three remarks should be noted: (1) An arbitrarily acoustic wave emitter situated in the near-field of the AHMM can be coupled to the high DOS modes of the AHMM efficiently. The high DOS modes in the AHMM are mainly made up of the superposition of different evanescent components with ${k}_{x}\gg {k}_{0}$ or ${k}_{x}\,\ll -\,{k}_{0}$ depending on the interface relation between the air and the AHMM $\,{k}_{{\rm{y}}}=i\sqrt{{k}_{{\rm{x}}}^{2}-{k}_{0}^{2}}\approx \pm \,i{k}_{{\rm{x}}}$, where the upper (lower) sign corresponds to ${k}_{{\rm{x}}}\gg {k}_{0}\,({k}_{{\rm{x}}}\ll -{k}_{0})$. (2) An acoustic dipole situated in the near-field of the AHMM excites the high DOS modes to the right and left equally. For example, for acoustic vertical dipole with the dipolar moment of ${\bf{p}}=[0,1]$, $P({k}_{{\rm{x}}},y)$ corresponding to ${k}_{{\rm{x}}}\gg {k}_{0}$ and ${k}_{{\rm{x}}}\ll -\,{k}_{0}$ are equal to each other [see Fig. 1(b)]. (3) The destructive interference condition applied to $y > {y}_{0}$ can be fulfilled if

3 $${p}_{{\rm{x}}}{k}_{{\rm{x}}}+{p}_{{\rm{y}}}{k}_{{\rm{y}}}={\rm{0}}$$

Thus, an acoustic helical wave emitter situated in the near-field of the AHMM results in the unidirectional excitation since it has the form $\frac{{p}_{{\rm{y}}}}{{p}_{{\rm{x}}}}=\pm \,i$. For example, for the acoustic counterclockwise helical wave emitter $p=[1,-\,i]$, Eq. (3) can be only fulfilled when ${k}_{x}\ll -\,{k}_{0}$, and thus unidirectional excitation is observed [see Fig. 1(c)]. The absolute pressure along the cutline *y* = 200 mm in the Fig. 1(b and c) verify the analysis demonstrated above [see Fig. 1(d)].

**Figure 2:**
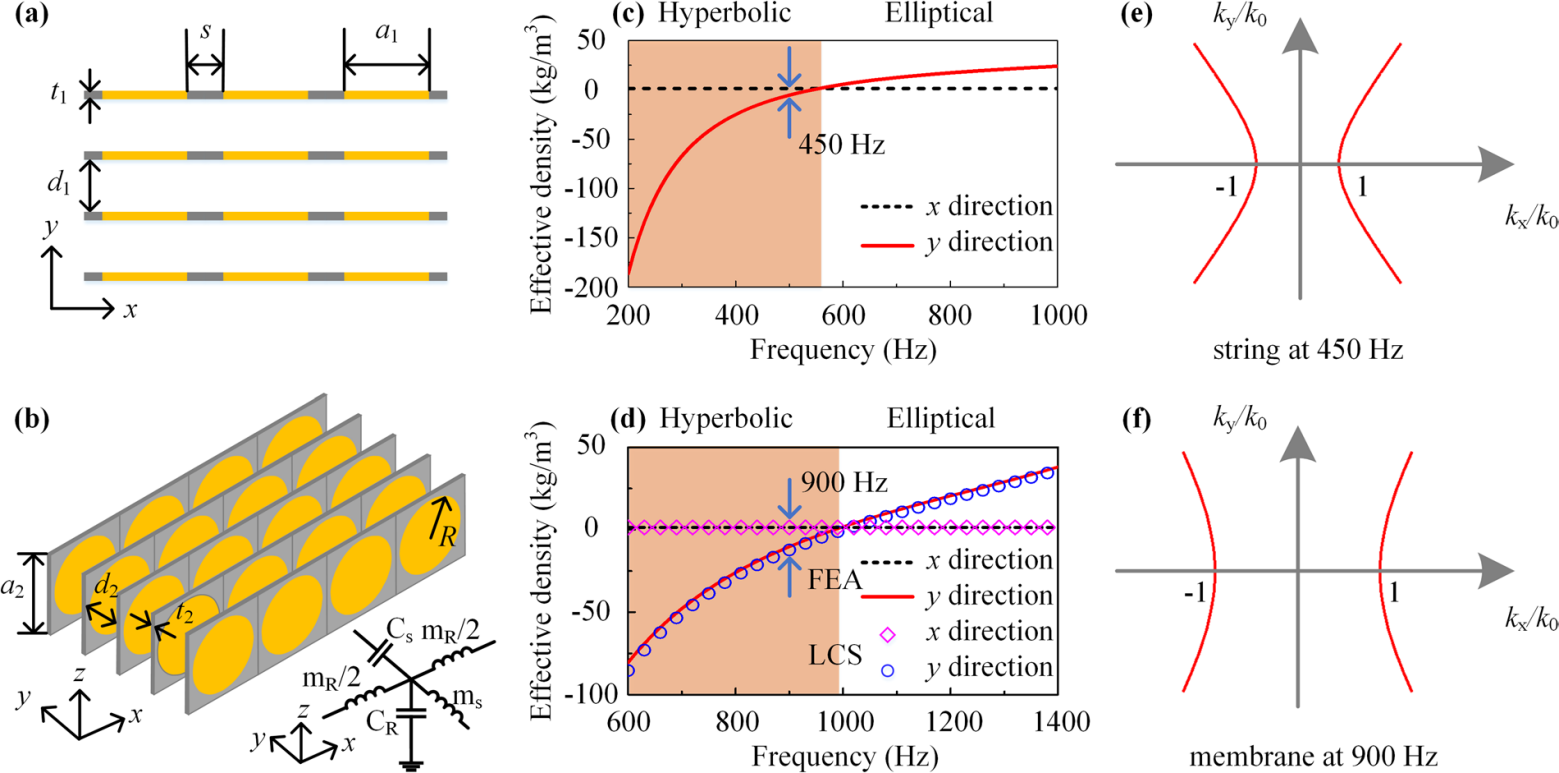
Designs of **(a)** the string-type and **(b)** the membrane-type AHMM. Retrieved effective mass density along the *x* and *y* directions of the **(c)** string-type and **(d)** membrane-type AHMM. The orange region corresponds to the frequency range of the hyperbolic dispersion (${\rho }_{x}\cdot {\rho }_{y}\, < \,0$). The EFC of **(e)** the string-type AHMM at 450 Hz and **(f)** membrane-type AHMM at 900 Hz.

We continue to demonstrate the physical prototypes of the AHMM. The string-type AHMM is illustrated in Fig. 2(a). Each of the two frames has a separation distance of ${d}_{1}={\rm{9.875}}\,{\rm{mm}}$, and the thickness of the frame is ${t}_{1}={\rm{0.125}}\,{\rm{mm}}$, and thus the frame in the *y* direction has a periodicity of ${D}_{1}={d}_{1}+{t}_{1}\,=\,{\rm{10}}\,{\rm{mm}}$. The widths of the frame and the string are $s\,=\,2\,{\rm{mm}}$ and ${a}_{1}={\rm{18}}\,{\rm{mm}}$, respectively. The design of the membrane-type AHMM is illustrated in Fig. 2(b). Each of the two frames has a separation distance of ${d}_{2}={\rm{9.875}}\,{\rm{mm}}$, and the thickness of the frame is ${t}_{2}={\rm{0.125}}\,{\rm{mm}}$, and thus the frame in the *y* direction has a periodicity of ${D}_{2}={d}_{2}+{t}_{2}={\rm{10}}\,{\rm{mm}}$. The radius of the membrane is $R=9\,{\rm{mm}}$, and the width of the square frame is ${a}_{2}={\rm{20}}\,{\rm{mm}}$. The string/membrane has a thickness of 0.125 mm. The string/membrane-type AHMM consists of 21 × 80 string/membranes unit cells, but there are only 4 × 3 string unit cells shown in Fig. 2(a) and 5 × 4 membrane unit cells in Fig. 2(b) for clarity. The frames are acoustically rigid. The strings/membranes are fixed securely on the rigid frames to achieve the clamped boundary condition. No tension is applied on the strings/membranes. Two rigid panels cover the top and bottom of the membrane-type AHMM to ensure 2D wave propagation.

We use the transmission line model to understand the proposed membrane-type AHMM, which can be represented by the 2D array of the unit structure [see the inset in Fig. 2(b)]. The voltage *U* at one node in the grid is related to its neighbors via the Kirchhoff current law^29^:

4 $${Y}_{{\rm{x}}}({U}_{{\rm{x}}-1,{\rm{y}}}+{U}_{{\rm{x}}+1,{\rm{y}}})+{Y}_{{\rm{y}}}({U}_{{\rm{x}},{\rm{y}}-1}+{U}_{{\rm{x}},{\rm{y}}+1})-2{U}_{{\rm{x}},{\rm{y}}}({Y}_{{\rm{x}}}+{Y}_{{\rm{y}}})\,=\,{U}_{{\rm{x}},{\rm{y}}}{Y}_{{\rm{z}}}$$

where *x* and *y* denote the node position, and ${Y}_{{\rm{x}}}$ and ${Y}_{{\rm{y}}}$ are the respective admittances. ${Y}_{{\rm{z}}}$ corresponds to the admittance of the shunt element. Equation (4) can be recast in a continuous form:

5 $${Y}_{{\rm{x}}}{\partial }_{{\rm{xx}}}\tilde{U}+{Y}_{{\rm{y}}}{\partial }_{{\rm{yy}}}\tilde{U}\,=\,\frac{1}{S}{Y}_{{\rm{z}}}\tilde{U},$$

where *S* is the area of the unit cell, and $\tilde{U}$ is the continuous counterpart of *U*. Considering ${Y}_{{\rm{x}}}\,=\,\frac{1}{i{\rm{\omega }}{L}_{{\rm{x}}}}$, ${Y}_{{\rm{y}}}\,=\,\frac{1}{i{\rm{\omega }}{L}_{{\rm{y}}}+1/(i{\rm{\omega }}{C}_{{\rm{y}}})}$, ${Y}_{{\rm{z}}}\,=\,i{\rm{\omega }}{C}_{{\rm{z}}}$, Eq. (5) can be recast as:

6 $$\frac{{k}_{{\rm{x}}}^{2}}{{L}_{{\rm{x}}}}+\frac{{k}_{{\rm{y}}}^{2}}{{L}_{{\rm{y}}}(1-\frac{1}{{\omega }^{2}{L}_{{\rm{y}}}{C}_{{\rm{y}}}})}\,=\,\frac{{\omega }^{2}}{S/{C}_{{\rm{z}}}}$$

It can be shown that ${L^{\prime} }_{{\rm{x}}}={L}_{{\rm{x}}}$, ${L^{\prime} }_{{\rm{y}}}={L}_{{\rm{y}}}({\rm{1}}-\frac{{\omega }_{c}^{2}}{{\omega }^{2}})$ and $C^{\prime} ={C}_{z}/S$, where ${\omega }_{c}\,=\,\sqrt{1/({L}_{{\rm{y}}}{C}_{{\rm{y}}})}$.

The similarity between the Eq. (6) and the dispersion relation implies the one-to-one correspondences for all quantities. The constitutive parameters ${L^{\prime} }_{{\rm{x}}}$ and ${L^{\prime} }_{{\rm{y}}}$ for the circuit corresponds to the effective mass density of the system and the $C^{\prime}$ corresponds to the bulk modulus. The effective density along the *x* direction remains the same as air, ${\rho ^{\prime} }_{{\rm{x}}}={\rho }_{{\rm{air}}}$, because there are no membrane arranged in the *x* direction. The effective bulk modulus $B^{\prime} ={B}_{{\rm{air}}}$, because the thin membrane has a negligible effect on the effective bulk modulus. From Eq. (6), it is noted that the effective density along the *y* direction ${\rho ^{\prime} }_{{\rm{y}}}=\rho ^{\prime} ({\rm{1}}-\frac{{\omega }_{c}^{2}}{{\omega }^{2}})$. Clearly, at a frequency below the first resonance frequency, the effective density along the *y* direction is negative, resulting in a broadband negative density. The membranes have a very good accuracy with a series resonant circuit with acoustic mass ${m}_{{\rm{am}}}$ and compliance ${C}_{{\rm{am}}}$ expressed by^30^:

7 $${m}_{{\rm{am}}}\,=\,{\rm{1.8830}}\frac{{\rho }_{{\rm{m}}}h}{\pi {R}^{2}},{C}_{{\rm{am}}}\,=\,\frac{\pi {R}^{6}}{{\rm{196.51}}D}$$

here, *h* is the thickness of the membrane, and $D\,=\,E{h}^{3}/\mathrm{12}(1-{\nu }^{2})$ represents its flexural rigidity. The parameters of the lumped element model in the *y* direction in the inset of Fig. 2(b) can be given by ${m}_{{\rm{s}}}\,=\,{m}_{{\rm{a}}}+{m}_{{\rm{am}}}$ and ${C}_{{\rm{s}}}\,=\,{C}_{{\rm{am}}}$, where ${m}_{{\rm{a}}}\,=\,{\rho }_{{\rm{air}}}(d-h)/S$ is the acoustic mass of the air section and *S* is the cross section area of the unit. The compliance of the air section is negligible since the size of the unit of the membrane-type AHMM is far less than the wavelength. The acoustic mass and the acoustic compliance of the lumped element model of air can be calculated according to the density (${\rho }_{{\rm{air}}}$) and the bulk modulus (${K}_{{\rm{air}}}$) of air such as ${m}_{{\rm{R}}}=\,{\rho }_{{\rm{air}}}d/{\rm{\Delta }}S$ and ${C}_{{\rm{R}}}\,=\,{\rm{\Delta }}S\times d/{K}_{{\rm{air}}}$, where $\Delta S$ is the area of the cross section. It follows that, the sound transmission in both the membrane network and the air can be simulated with the parameters demonstrated above. The parameters of the membrane network can be derived that ${Y}_{{\rm{x}}}\,=\,\frac{1}{i{\rm{\omega }}{L}_{{\rm{x}}}}\,=\,\frac{1}{i{\rm{\omega }}{m}_{{\rm{R}}}}$, ${Y}_{{\rm{y}}}\,=\,\frac{1}{i{\rm{\omega }}{m}_{{\rm{s}}}+1\,/\,(i{\rm{\omega }}{C}_{{\rm{s}}})}$, ${Y}_{{\rm{z}}}\,=\,i{\rm{\omega }}{C}_{{\rm{R}}}$. In order to verify the broadband negative density of the AHMMs, a rigorous retrieving method is used to obtain its effective parameters^31^. The effective density of a unit cell in the string/membrane-type AHMM is negative over the hyperbolic frequency band [see Fig. 2(c,d)]. The dispersion is clearly hyperbolic below the first resonance frequency [see Fig. 2(e,f)].

**Figure 3:**
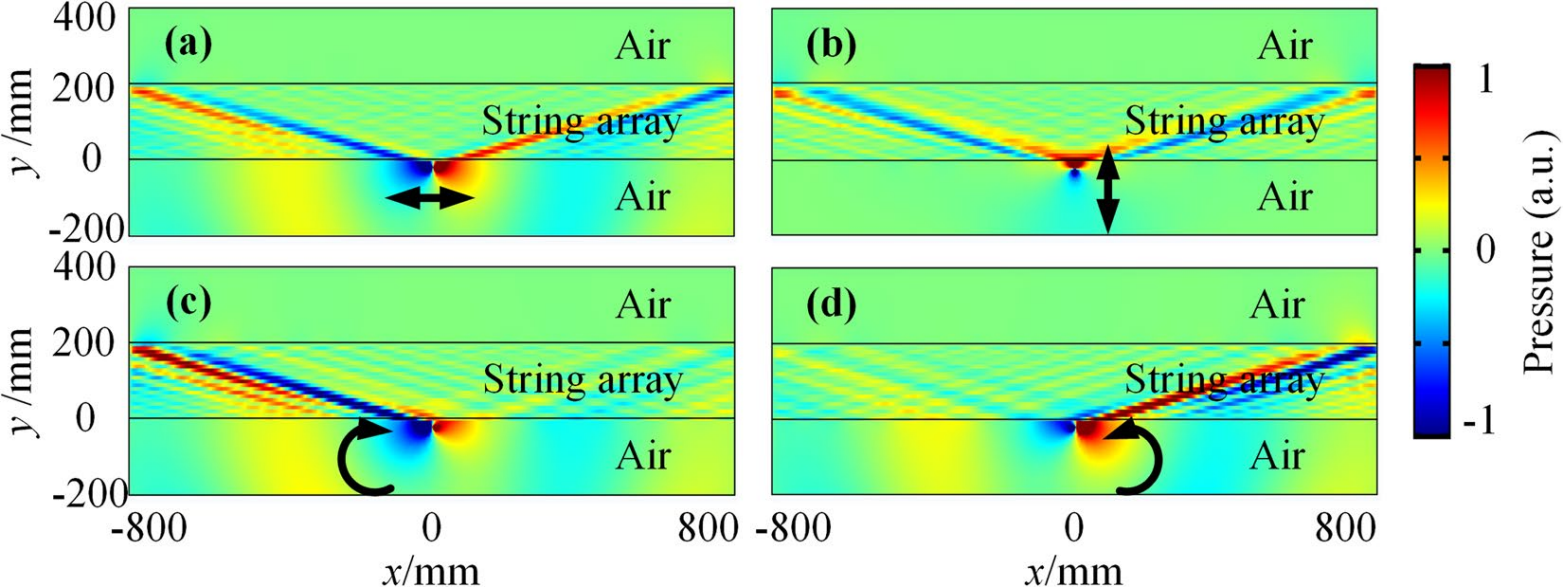
ASHE in the string-type AHMM. The pressure field excited by **(a)** the acoustic horizontal dipole $p\,=\,[1,0]$, **(b)** the acoustic vertical dipole $p\,=\,[0,1]$, **(c)** the acoustic clockwise helical wave emitter $p\,=\,[1,i]$ and **(d)** the acoustic counterclockwise helical wave emitter $p\,=\,[1,-\,i]$ situated in the near-field of the string-type AHMM (See Supplementary Movie 2).

In order to present the ASHE in the prototypes of proposed AHMMs, the full-wave numerical simulations are performed. Figure 3 shows the pressure field when the acoustic dipole or the helical wave emitter is in near-field of the string-type AHMM. The numerical simulation is realized at 450 Hz where the dispersion of the string-type AHMM is hyperbolic according to Fig. 2(e). The boundary of the domain is set to be radiative and the acoustic wave emitter in air is situated 20 mm away from the string array. Both the acoustic horizontal and vertical dipoles excite the high DOS modes equally to the right and left from its position [see Fig. 3(a,b)]. The acoustic helical wave emitter is shown to support the unidirectional excitation [see Fig. 3(c,d)], which is in good agreement with the destructive interference condition (Eq. (3)). Changing the helical direction of the helical wave switches the direction of the emission. The phenomenon in Fig. 3 has proved the ASHE in the string-type AHMM. A 16 dB power ratio radiated in different directions is found for acoustic clockwise and counterclockwise helical wave emitters and the string-type AHMM is shown to support the strongly confined subwavelength modes with about $\lambda /8$ full width at half maximum (FWHM). Since the only requirement on the helical-wave-induced routing by the metamaterial is its hyperbolic dispersion, the reported unidirectional excitation is inherently broadband for the string-type AHMM.

**Figure 4:**
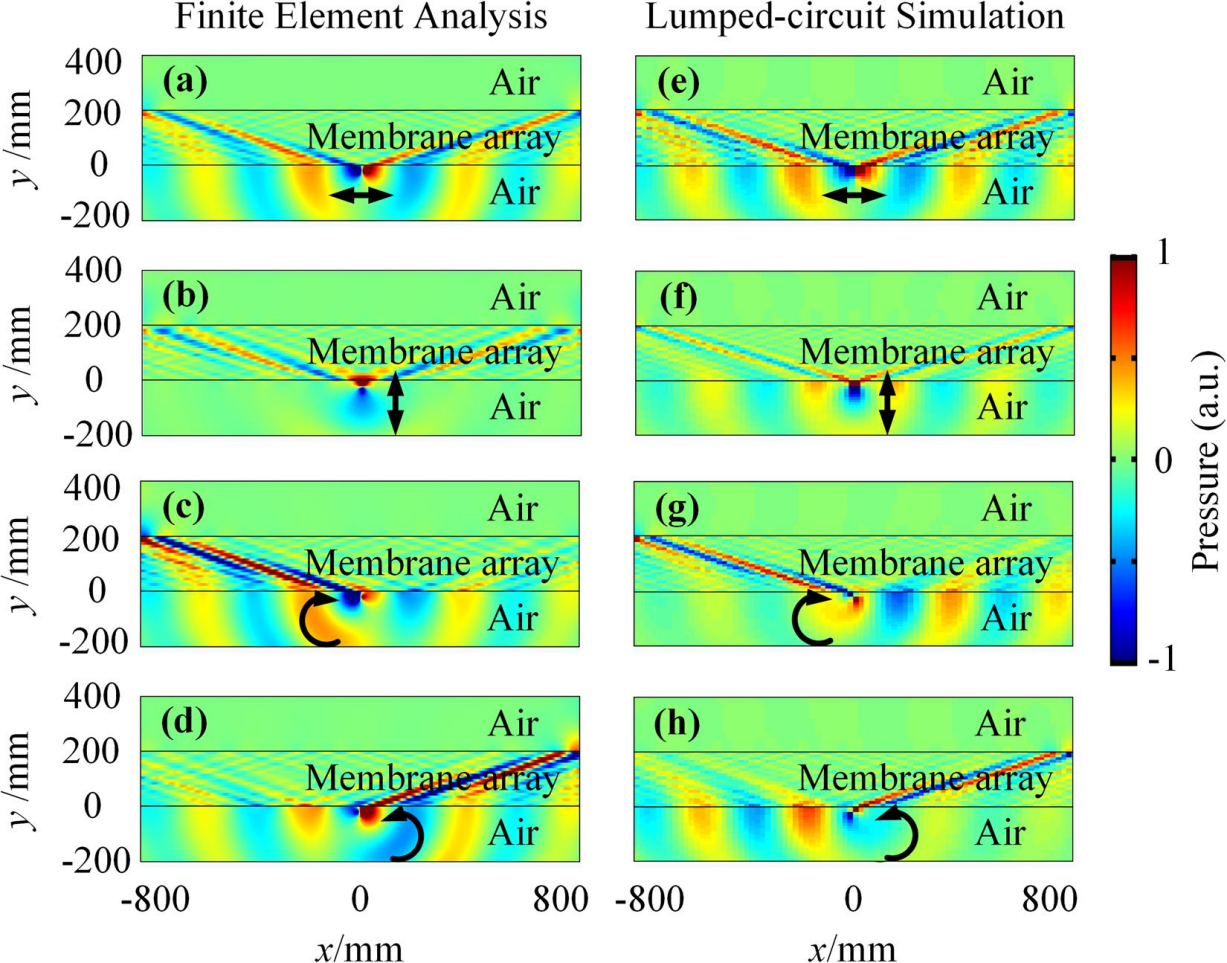
ASHE in the membrane-type AHMM. The pressure field excited by **(a)** the acoustic horizontal dipole $p\,=\,[1,0]$, **(b)** the acoustic vertical dipole $p\,=\,[0,1]$, **(c)** the acoustic clockwise helical wave emitter $p\,=\,[1,i]$ and **(d)** the acoustic counterclockwise helical wave emitter $p\,=\,[1,-\,i]$ situated in the near-field of the membrane-type AHMM. **(e–h)** Corresponding lumped-circuit simulation results (See Supplementary Movie 3).

Furthermore, in terms of the similar properties of the membrane and the string, the membrane-type AHMM is also demonstrated to enable the ASHE, which can be realized more straightforward. Figure 4 shows the pressure field when the acoustic dipole or the helical wave emitter is in near-field of the membrane-type AHMM through three-dimensional (3D) full-wave acoustic simulations as well as Lumped-circuit (LC) simulations. Similar to the ASHE in the string-type AHMM, while an acoustic horizontal or vertical dipole excites equally the high DOS modes to the right and left from its position [see Fig. 4(a,b)], the acoustic helical wave emitter destructively interferes in one direction [see Fig. 4(c,d)] depending on the helical directions. In the LC simulations, the metamaterial slab is represented by periodically repeating the unit cell in the inset of Fig. 2(b). The LC simulation results [see Fig. 4(e–h)] show good agreement with the corresponding acoustic simulation results [see Fig. 4(a–d)]. The phenomenon in Fig. 4 has proved the ASHE in the membrane-type AHMM. A 14 dB power ratio radiated in different directions is found for acoustic clockwise and counterclockwise helical wave emitters and the membrane-type AHMM is shown to support the strongly confined subwavelength modes with about $\lambda /6$ FWHM. The reported unidirectional excitation is inherently broadband for the membrane-type AHMM too.

## Discussion

The ASHE in the string-type AHMM and the membrane-type AHMM has been observed. The ASHE has a remarkably close analogy to the SHE for electrons and the photonic spin Hall effect. In our realization, the ASHE effect is observed due to the inversion symmetry broken by the acoustic helical wave emitter. The directional broadband excitation of guided modes is achieved with an acoustic helical wave emitter, which is in close analogy to the electron sorting by their spins in a current flow and the unidirectional emitting of photons with different polarizations. The emergence of the phenomenon was numerically demonstrated with the string-type AHMM at 450 Hz and the membrane-type AHMM at 900 Hz. The experiment of the ASHE in the membrane-type AHMM is believed to be implemented where the membrane array is conducted by thick plastic walls with round openings, and polyethylene film attached airtight to the rims^32^. The other point of the experiment is the acoustic helical emitter, which can be realized with four point sources with phase step of ${\rm{\pi }}/2$. The applications of the demonstrated phenomenon are not limited in unidirectional excitation. The proposed ASHE can also be applied to detect the orbital angular momentum of the acoustic waves and provide a possibility to the new quantum-like effect of the acoustic chirality.

## Methods

Throughout the paper, the numerical simulations of Finite Element Analysis (FEA) are performed by the commercial finite element package COMSOL Multiphysics. The mass density (${\rho }_{{\rm{m}}}$), Young’s modulus (*E*), and Poisson’s ratio ($\nu$) of the string/membrane are 1420 kg/m^3^, 2.5 GPa, and 0.34, respectively. The mass density and the bulk modulus of air are ${\rho }_{{\rm{air}}}={\rm{1.39}}\,{\mathrm{kg}/{\rm{m}}}^{3}$ and ${K}_{{\rm{air}}}={\rm{152}}\,{\rm{KPa}}$, respectively. The parameters are identical with the ref 27^33^. The Lumped-circuit simulations (LCS) are performed by the Advanced Design System. The parameters in the LCS can be retrieved by the transmission line model demonstrated above.

### Data availability

The datasets generated during and/or analyzed during the current study are available from the corresponding author on reasonable request.

## Electronic supplementary material

Supplementary material

Supplemental Movie 1

Supplemental Movie 2

Supplemental Movie 3
