# Supplementary material for: Acoustic spin Hall-like effect in hyperbolic metamaterials controlled by the helical wave
Source: Sci Rep. 2018 Jul 24;8:11113. doi: 10.1038/s41598-018-29359-w (PMC6057999; doi:10.1038/s41598-018-29359-w)
Supplement: Supplementary file 1 — Supplementary material [file 41598_2018_29359_MOESM1_ESM.pdf]

Supplementary material

Acoustic spin Hall-like effect in hyperbolic metamaterials controlled by the helical wave

Fangfang Ju[1], Ying Cheng[1,2*], Xiaojun Liu[1,2†]

[1]Key Laboratory of Modern Acoustics, Department of Physics and Collaborative Innovation Center of Advanced Microstructures, Nanjing University, Nanjing 210093, China

[2]State Key Laboratory of Acoustics, Institute of Acoustics, Chinese Academy of Sciences, Beijing 100190, China

Correspondence and requests for materials should be addressed to Y.C. (email: chengying@nju.edu.cn) or X.J.L. (email: liuxiaojun@nju.edu.cn)

Movie legends of the supplementary movie

Movie 1. Dynamic view of the pressure field excited by **(a)** the acoustic horizontal dipole $\mathbf{p} = [1,0]$, **(b)** the acoustic vertical dipole $\mathbf{p} = [0,1]$, **(c)** the acoustic clockwise helical wave emitter $\mathbf{p} = [1,i]$ and **(d)** the acoustic counterclockwise helical wave emitter $\mathbf{p} = [1,-i]$ situated in the near-field of the ideal AHMM.

Movie 2. Dynamic view of the pressure field excited by **(a)** the acoustic horizontal dipole $\mathbf{p} = [1,0]$, **(b)** the acoustic vertical dipole $\mathbf{p} = [0,1]$, **(c)** the acoustic clockwise helical wave emitter $\mathbf{p} = [1,i]$ and **(d)** the acoustic counterclockwise helical wave emitter $\mathbf{p} = [1,-i]$ situated in the near-field of the string-type AHMM.

Movie 3. Dynamic view of the pressure field excited by **(a)** the acoustic horizontal dipole

$\mathbf{p} = [1,0]$, **(b)** the acoustic vertical dipole $\mathbf{p} = [0,1]$, **(c)** the acoustic clockwise helical wave emitter $\mathbf{p} = [1,i]$ and **(d)** the acoustic counterclockwise helical wave emitter $\mathbf{p} = [1,-i]$ situated in the near-field of the membrane-type AHMM.
